# Effect of erythropoietin on the incidence of acute kidney injury following complex valvular heart surgery: a double blind, randomized clinical trial of efficacy and safety

**DOI:** 10.1186/cc13081

**Published:** 2013-10-24

**Authors:** Ji-Ho Kim, Jae-Kwang Shim, Jong-Wook Song, Young Song, Hye-Bin Kim, Young-Lan Kwak

**Affiliations:** 1 Department of Anesthesiology and Pain Medicine, National Health Insurance Service Ilsan Hospital, Gyeonggi-do, South Korea; 2 Department of Anesthesiology and Pain Medicine, Anesthesia and Pain Research Institute, Yonsei University Health System, Seoul, South Korea; 3 Biomedical Science Institute, Department of Anesthesiology and Pain Medicine, Anesthesia and Pain Research Institute, Yonsei University Health System, 250 Seongsanno, Sedaemun-gu, Seoul 120-752, South Korea

## Abstract

**Introduction:**

Recombinant human erythropoietin (EPO) is known to provide organ protection against ischemia-reperfusion injury through its pleiotropic properties. The aim of this single-site, randomized, case-controlled, and double-blind study was to investigate the effect of pre-emptive EPO administration on the incidence of postoperative acute kidney injury (AKI) in patients with risk factors for AKI undergoing complex valvular heart surgery.

**Methods:**

We studied ninety-eight patients with preoperative risk factors for AKI. The patients were randomly allocated to either the EPO group (n = 49) or the control group (n = 49). The EPO group received 300 IU/kg of EPO intravenously after anesthetic induction. The control group received an equivalent volume of normal saline. AKI was defined as an increase in serum creatinine >0.3 mg/dl or >50% from baseline. Biomarkers of renal injury were serially measured until five days postoperatively.

**Results:**

Patient characteristics and operative data, including the duration of cardiopulmonary bypass, were similar between the two groups. Incidence of postoperative AKI (32.7% versus 34.7%, *P* = 0.831) and biomarkers of renal injury including cystatin C and neutrophil gelatinase-associated lipocalin showed no significant differences between the groups. The postoperative increase in interleukin-6 and myeloperoxidase was similar between the groups. None of the patients developed adverse complications related to EPO administration, including thromboembolic events, throughout the study period.

**Conclusions:**

Intravenous administration of 300 IU/kg of EPO did not provide renal protection in patients who are at increased risk of developing AKI after undergoing complex valvular heart surgery.

**Trial registration:**

Clinical Trial.gov, NCT01758861

## Introduction

Despite advances in the understanding of its pathophysiology, acute kidney injury (AKI) continues to be a serious complication following cardiac surgery [1]. AKI is an independent predictor of both in-hospital and long-term mortality in cardiac surgical patients [2]. Of note, even a minor increase in serum creatinine (sCr) level is associated with increased risk of morbidity and mortality, not to mention that the most severe disease spectrum of AKI requires dialysis [3,4].

Patient-related factors and cardiopulmonary bypass (CPB) are considered major risk factors for AKI [5]. CPB is associated with inevitable alterations contributing to the development of AKI, such as ischemia-reperfusion injury, systemic inflammatory response, hemodilution, and embolism [6]. Indeed, the reported incidence of AKI after cardiac surgeries that required prolonged durations of CPB reaches 40% to 50%, depending on the chosen definition [7]. Considering its high prevalence and clear association with poor prognosis, the development of renal protective strategies is important, especially in patients with known risk factors for AKI undergoing surgeries requiring prolonged durations of CPB.

Erythropoietin (EPO) is a natural hormone produced mainly in the kidney that regulates hematopoiesis. Apart from its hematopoietic effect, recent literature supports a protective role against ischemia-reperfusion injury through its anti-oxidative, anti-inflammatory and anti-apoptotic effects [8-10]. Indeed, EPO has been shown to decrease myocardial and renal damage after hypoxic insult in numerous experimental studies; however, conflicting results have been reported in clinical studies [11-13]. Despite its extensive experimental background, evidence is lacking regarding EPO’s renal protective effect in cardiac surgical patients who are at increased risk of developing AKI.

The aim of this single-site, randomized, controlled and double-blind study is to validate whether a pre-emptive single bolus of recombinant human EPO mitigates the occurrence of AKI following complex valvular heart surgery in patients with preoperative risk factors for AKI.

## Materials and methods

### Patients

After approval by the institutional review board of the Severance Hospital, Yonsei University Health System, Seoul, Korea, and procurement of informed consent, we studied ninety-eight patients with preoperative risk factors for AKI who were scheduled for complex valvular heart operations. This single-site trial was conducted at Yonsei University Health System, Seoul, Korea (ClinicalTrials.gov NCT0178861). Enrollment criteria for high risk of AKI included more than two of the following: female, chronic obstructive pulmonary disease, preoperative creatinine >1.2 mg/dl, New York Heart Association functional class IV, left ventricle ejection fraction <35%, diabetes mellitus (DM), age >65 years or peripheral vascular disease [5]. Complex valvular heart operations were defined as double-valve surgery, combined valve and coronary artery bypass grafting procedures, Bentall operations, combined mitral valve surgery and tricuspid annuloplasty or repeat operations. Patients with pre-existing uncontrolled hypertension (diastolic blood pressure >100 mmHg), immunosuppression, history of thromboembolism, malignant disease, seizure, liver dysfunction, end-stage renal disease requiring renal replacement therapy and drug or alcohol abuse were excluded.

### Treatment

Patients were randomly allocated to either the EPO or control group in a 1:1 ratio according to simple randomization using a computer-generated random code. Patients in the EPO group received 300 IU/kg of human recombinant EPO-alpha (Epocain; CJ Pharma, Seoul, Korea) intravenously after anesthetic induction. Patients in the control group received an equal volume of normal saline at the same time point. Medications were prepared by a nurse who knew the patient’s group assignment but was not involved in the study.

### Clinical evaluations

The primary endpoint of interest was incidence of AKI, which was diagnosed according to the Acute Kidney Injury Network (AKIN) criteria (absolute increase in the sCr concentration ≥0.3 mg/dL from baseline or ≥50% increase in the sCr concentration within 48 hours after operation) [14].

The secondary outcomes assessed in this study were biomarkers of renal injury including sCr, cystatin C, and estimated glomerular filtration rate (eGFR) which were recorded 24 hours before operation, upon arrival at the ICU, and on postoperative days (POD) 1, 2, 3, and 5. Additionally, changes in serum IL-6, myeloperoxidase (MPO) and neutrophil gelatinase-associated lipocalin (NGAL) levels in 18 randomly selected patients from each group were measured after anesthetic induction, at 1 and 6 hours after removal of the aortic cross clamp (ACC), and 24 hours after operation. Quantikine® human IL-6 and Quantikine® human MPO (R&D System Inc., Minneapolis, MN, USA) were used to measure IL-6 and MPO. NGAL was measured by Human Lipocalin-2/NGAL Immunoassay (R&D System Inc., Minneapolis, MN, USA).

Preoperative variables included demographic data, type of operation and co-morbid conditions such as DM, hypertension, chronic kidney disease, cerebrovascular accidents, congestive heart failure, medications and EuroSCORE.

Intraoperative variables included operation time, duration of ACC and CPB, fluid balance, and packed erythrocyte transfusion requirement. The amount of ultrafiltration during CPB and the amount of salvaged blood by a cell salvage device during the operation were also recorded.

Postoperative variables included packed erythrocyte transfusion requirement, fluid balance and bleeding measured by chest tube drainage during the 24 hours after the operation. Hematocrit was measured preoperatively and on POD 0, 1, 2, 3, and 5. Other postoperative variables were length of ICU and hospital stay, prolonged mechanical ventilator care (>48 hours), inotrope use, re-intubation, re-operation, atrial fibrillation, stroke and in-hospital mortality. Possible complications associated with EPO treatment, such as hypertension, headache, tachycardia, nausea, vomiting, diarrhea and thromboembolism, were also assessed during the study period.

### Perioperative management

All patients received standard anesthetic care. Standard monitoring devices included a pulmonary artery catheter and transesophageal echocardiography. Mechanical ventilation was controlled to maintain normocarbia with a tidal volume of 8 mL/kg and respiratory rate of 8 to 14 breaths/minute.

All patients received standard surgical and CPB management with ultrafiltration. CPB was instituted with a membrane oxygenator primed with 1.6 L of priming solution. Non-pulsatile pump flow was maintained at a rate of 2.0 to 2.4 L/min/m^2^. During the intraoperative period, including CPB, mean arterial pressure was maintained at 50 to 80 mmHg with norepinephrine or sodium nitroprusside.

During the perioperative period, fluid balance was maintained using Voluven® (Fresenius Kabi, Graz, Austria) and Plasma solution A 1000 inj® (CJ Pharma, Seoul, Korea) to maintain normovolemic status. Total volume of intraoperative Voluven® was limited to less than 20 ml/kg and/or 1,000 ml. The threshold for transfusion of packed erythrocytes was hematocrit <20% during CPB or <25% after CPB. After heparin was antagonized with protamine, blood from the CPB circuit was salvaged using a cell salvage device and re-transfused into all patients.

For postoperative hemodynamic management, norepinephrine or nicardipine was used to maintain mean arterial pressure between 65 to 90 mmHg. As an inotrope, milrinone was used if the left ventricular ejection fraction was lower than 30% or in cases of right ventricular dysfunction and/or pulmonary hypertension. Diuretics were used when hourly urine output was less than 0.5 ml/kg. Blood glucose levels were targeted between 100 and 250 mg/dL with insulin throughout the study period.

### Statistical analysis

Data were analyzed with SPSS version 19.0 statistical software (SPSS INC, an IBM Company, Chicago, IL, USA) and SAS version 9.2 (SAS Institute Inc., Cary, NC, USA). Results are expressed as mean ± SD, median (interquartile ranges (25th to 75th)) or number of patients (percentages). Continuous variables were compared using independent Student’s *t*-test or Mann–Whitney *U* test, and dichotomous variables were compared using chi-square or Fisher’s exact test as appropriate. The Shapiro-Wilk test for normality was performed. NGAL and inflammatory cytokine levels were log-transformed. Repeatedly measured variables, such as creatinine, cystatin C, eGFR, log NGAL, log IL-6 and log MPO were analyzed using a linear mixed model as a group, time and group-by-time as fixed effects. Type I error was corrected by the Bonferroni method. All statistical tests were two-tailed. *P*-value less than 0.05 was considered statistically significant. In a previous institutional study, the incidence of AKI following valvular heart surgery in anemic patients was 54%, [13] and EPO reduced the incidence of AKI more than 50% in two previous clinical studies [11,13]. Therefore, we determined that 49 patients were required in each group to detect a 50% reduction in the incidence of AKI between the groups to obtain a power of 80%, considering a type I error of 0.05. Regarding the measurement of NGAL, we determined that 18 patients were required in each group to detect a mean difference of 50 ng/ml (SD of 50 ng/ml) to obtain a power of 80%, considering a type I error of 0.05 [15,16].

## Results

**Figure 1:**
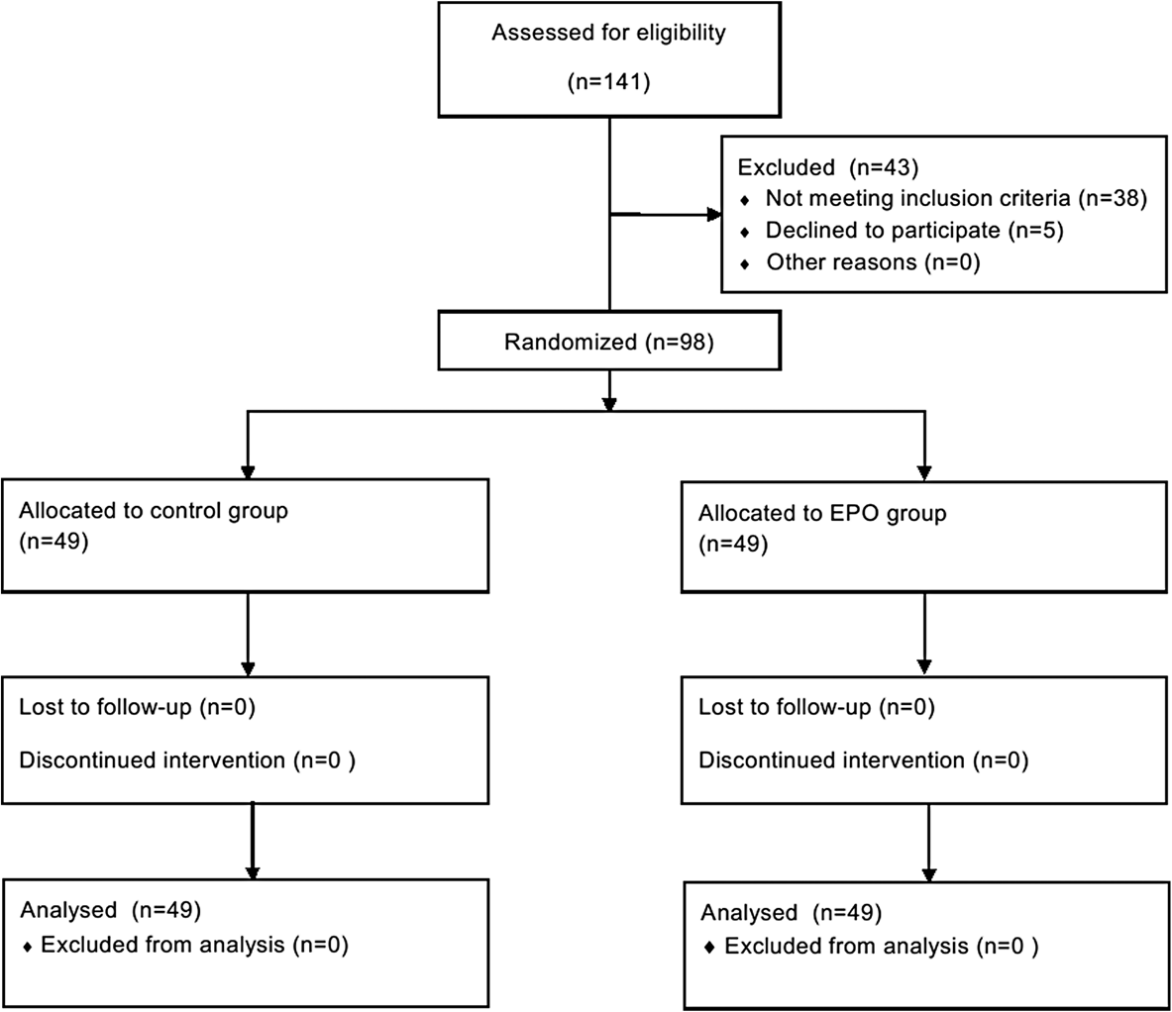
Diagram representing study cohort allocation.

A total of ninety-eight patients were randomized to receive EPO (n = 49) or normal saline (n = 49), and no patient was dropped out after group allocation (Figure 1). Patients’ characteristics, including demographic data, EuroSCORE and preoperative renal function were not statistically different between the two groups (Table 1).

**Table 1:** Patients’ demographics and preoperative clinical data

**Variable**	**Control (number = 49)**	**EPO (number = 49)**	***P *** **value**
Age (years)	62 ± 10	63 ± 10	0.880
Female	24 (49)	20 (41)	0.417
BMI (kg/m^2^)	24 ± 4	24 ± 3	0.528
Operation			
MVR + TAP	6 (12)	8 (16)	0.564
Multi valve	22 (45)	20 (41)	0.683
Valve + CABG	8 (16)	7 (14)	0.779
Bentall	13 (27)	14 (29)	0.821
Redo	6 (12)	4 (8)	0.505
NYHA			
I-II	42 (86)	43 (88)	0.766
III-IV	7 (14)	6 (12)	
EuroSCORE	5.1 ± 2.6	5.7 ± 2.5	0.299
DM	8 (16)	16 (33)	0.060
Hypertension	24 (49)	27 (55)	0.544
Chronic kidney disease	14 (29)	10 (20)	0.347
Chronic obstructive lung disease	2 (4)	0 (0)	0.495
Ejection fraction (%)	60 ± 13	60 ± 12	0.798
Preoperative eGFR	77 ± 30	83 ± 28	0.378
Preoperative CrCl <60 ml/min	21 (43)	17 (35)	0.407
Medication			
β-blockers	18 (38)	14 (29)	0.350
CCB	17 (35)	13 (27)	0.344
ACEi	17 (35)	12 (25)	0.240
ARB	16 (33)	13 (27)	0.464
Diuretics	28 (58)	28 (57)	0.906
Preoperative hematocrit (%)	38 ± 6	39 ± 4	0.375

Values are mean ± SD or number of patients (%). ACEi, angiotensin-converting-enzyme inhibitors; ARB, angiotensin II receptor blockers; BMI, body mass index; CCB, calcium channel blockers; CrCl, creatinine clearance; DM, diabetes mellitus; eGFR, estimated glomerular filtration rate; EPO, erythropoietin; Multi valve, more than two valve replacement; MVR, mitral valve replacement; NYHA, New York Heart Association Functional Classification; TAP, tricuspid annuloplasty; Valve + CABG, valvular replacement with coronary artery bypass grafting.

Operative characteristics, including the type of surgery performed, operation time, duration of CPB and ACC were similar between the two groups. Fluid balance, transfusion requirement, amount of ultrafiltration, re-infused volume from the cell savage device, vasopressor and inotropic requirement, and postoperative blood loss as measured by the volume of chest tube drainage were also similar between the two groups throughout the study period (Tables 2 and 3).

**Table 2:** Intraoperative parameters and fluid balance

**Variable**	**Control (number = 49)**	**EPO (number = 49)**	***P *** **value**
Operation time (min)	253 ± 68	243 ± 63	0.480
CPB time (min)	129 ± 42	128 ± 39	0.870
ACC time (min)	96 ± 38	96 ± 30	0.894
Norepinephrine infused (μg)	70 (40 to 117)	68 (36 to 101)	0.350
Amount of ultrafiltration (ml)	1,000 (563 to 1,575)	1,000 (450 to 2,000)	0.936
Fluid balance during operation			
crystalloid (ml)	1,570 ± 531	1,579 ± 487	0.934
colloid (ml)	500 (350 to 600)	450 (300 to 575)	0.398
urine output (ml)	1,022 ± 493	1,004 ± 505	0.863
Amount of pRBC transfusion (ml)	219 ± 384	122 ± 162	0.107
Amount of cell salvaged blood (ml)	550 (490 to 765)	500 (480 to 748)	0.268
Patients transfused with pRBCs (number)	20 (41)	21 (43)	0.838

Values are mean ± SD or median (25th to 75th). ACC, aorta cross clamp; CPB, cardiopulmonary bypass; EPO, erythropoietin; pRBCs, packed red blood cells.

**Table 3:** Postoperative care at ICU

**Variable**	**Control (number = 49)**	**EPO (number = 49)**	***P *** **value**
Crystalloid infused (ml)			
at ICU POD 0	2795 ± 994	3045 ± 1054	0.231
at ICU POD 1	2380 ± 695	2261 ± 861	0.452
Colloid infused (ml)			
at ICU POD 0	280 (100 to 500)	150 (0 to 500)	0.139
at ICU POD 1	0 (0 to 0)	0 (0 to 0)	0.317
Urine output (ml)			
at ICU POD 0	2,180 ± 701	2,389 ± 832	0.182
at ICU POD 1	2,520 ± 722	2,628 ± 537	0.401
pRBCs transfused (ml)			
at ICU POD 0	223 ± 344	222 ± 601	0.987
at ICU POD 1	63 ± 182	44 ± 136	0.555
Chest tube drainage (ml)			
at ICU POD 0	390 (195 to 580)	330 (195 to 595)	0.394
at ICU POD 1	240 (165 to 375)	270 (180 to 440)	0.153
Patients transfused with pRBCs (number) at POD 0 and 1	16 (33)	23 (47)	0.149
Patients treated with vasopressors (number) at POD 0 and 1	18 (37)	12 (25)	0.188
Patients treated with inotropics (number) at POD 0 and 1	21 (43)	17 (35)	0.407

Values are mean ± SD or median (25th to 75th). ACC, aorta cross clamp; CPB, cardiopulmonary bypass; EPO, erythropoietin; POD, postoperative day; pRBCs, packed red blood cells.

Overall, AKI occurred in 33 of 98 patients (34%). The incidence of AKI was similar between the groups. Among the patients who developed AKI, a similar number of patients required renal replacement therapy during the hospital stay. There were no statistically significant differences in sCr levels, eGFR, creatinine clearance, cystatin C levels or serum NGAL levels between the two groups. The highest sCr level was observed at POD 1 in both groups (Table 4).

**Table 4:** Variables associated with renal outcomes

**Variable**	**Control (number = 49)**	**EPO (number = 49)**	***P *** **value**
Postoperative AKI	16 (0.33, 0.26 to 0.39)	17 (0.35, 0.28 to 0.42)	0.831
Stage 1	12 (0.25, 0.18 to 0.31)	12 (0.25, 0.18 to 0.31)	1.000
Stage 2	0	0	1.000
Stage 3	4 (0.08, 0.04 to 0.12)	5 (0.10, 0.06 to 0.15)	0.727
Postoperative dialysis	4 (0.08, 0.04 to 0.12)	5 (0.10, 0.06 to 0.15)	0.727
Serum creatinine (mg/dl)			0.090^b^
Baseline	0.99 ± 0.27	0.92 ± 0.28	0.218
ICU arrival	0.89 ± 0.25^a^	0.92 ± 0.34	0.546
POD 1	1.13 ± 0.39^a^	1.08 ± 0.46^a^	0.588
POD 2	1.09 ± 0.55	1.07 ± 0.50a	0.795
POD 3	1.00 ± 0.54	0.96 ± 0.52	0.754
POD 5	0.87 ± 0.35^a^	0.85 ± 0.52	0.934
Cystatin C (mg/L)			0.423^b^
Baseline	1.13 ± 0.37	1.04 ± 0.28	0.191
ICU arrival	1.00 ± 0.27^a^	0.95 ± 0.31	0.457
POD 1	1.08 ± 0.36	1.09 ± 0.39	0.912
POD 2	1.30 ± 0.54^a^	1.26 ± 0.57^a^	0.736
POD 3	1.58 ± 0.89^a^	1.54 ± 0.88^a^	0.898
eGFR (mL/min/1.73 m^2^)			0.759^b^
Baseline	77 ± 30	83 ± 28	0.381
ICU arrival	89 ± 36^a^	94 ± 46^a^	0.556
POD 1	80 ± 46	79 ± 43	0.941
POD 2	72 ± 36	71 ± 30^a^	0.985
POD 3	79 ± 35	76 ± 30	0.659
POD 5	88 ± 32^a^	89 ± 30^a^	0.863
Log NGAL			0.891^b^
Baseline	1.53 ± 0.28	1.56 ± 0.26	0.780
1 hourr after ACC off	2.21 ± 0.26^a^	2.16 ± 0.28^a^	0.591
6 hourr after ACC off	2.24 ± 0.33^a^	2.17 ± 0.33^a^	0.550
24 hourr after operation	2.17 ± 0.37^a^	2.14 ± 0.36^a^	0.846

Values are number of patients (probability, 95% confidence interval) or mean ± SD. ^a^*P* <0.05, compared with baseline; ^b^*P*_group x time_ = *P*-value of the group and time interaction obtained by the linear mixed model. ACC, aortic cross clamp; AKI, acute kidney injury; eGFR, estimated glomerular filtration rate; EPO, erythropoietin; Log NGAL, logarithm of neutrophil gelatinase-associated lipocalin; POD, postoperative days.

**Figure 2:**
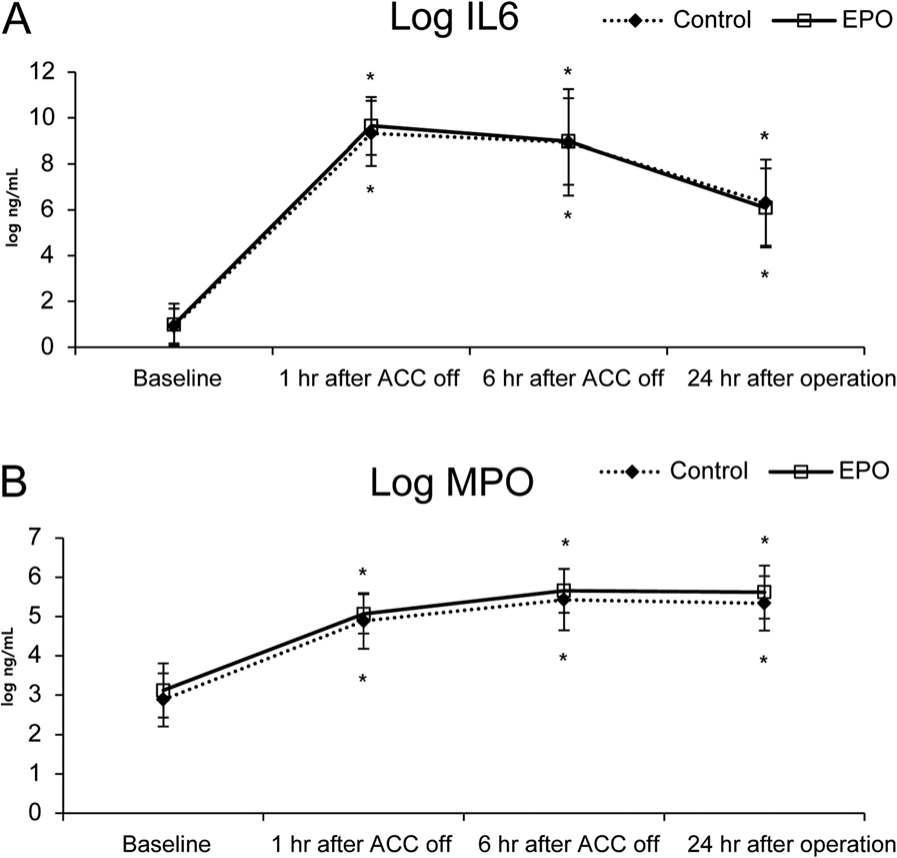
**Changes of log transformed serum interlukin-6 (A) and myeloperoxidase (B) over time in the control group and EPO group.** Values are mean ± SD. EPO, erythropoietin; Log IL6, logarithm of interlukin 6; Log MPO, logarithm of myeloperoxidase. *P <0.05, compared with baseline (type I error was corrected by Bonferroni method, all time points were statistically significantly different from baseline).

IL-6 and MPO were statistically significantly increased from their corresponding baseline values after the operation in both groups without any intergroup differences (Figure 2).

Perioperative hemodynamic variables, including mean arterial pressure, heart rate, central venous pressure, pulmonary capillary wedge pressure and cardiac index, were not different between the two groups (data not shown). Pre- and postoperative hematocrit levels were also similar between the two groups (Table 5).

**Table 5:** Changes of hematocrit during perioperative period

**Group**	**Preoperative**	**POD 0**	**POD 1**	**POD 2**	**POD 3**	**POD 5**
Control (n = 49)	38 ± 6	31 ± 4	29 ± 3	28 ± 3	27 ± 3	27 ± 3
EPO (n = 49)	39 ± 4	31 ± 5	29 ± 4	28 ± 4	28 ± 4	29 ± 3
*P* value	0.375	0.756	0.227	0.369	0.192	0.131

Values are mean ± SD. EPO, erythropoietin; POD, postoperative days.

The lengths of ICU and hospital stays were similar between the two groups. Remaining postoperative outcome variables were not different between the groups. One patient in the control group died during the hospital stay due to sepsis and acute respiratory distress syndrome (Table 6).

**Table 6:** Postoperative outcome

**Variable**	**Control (number = 49)**	**EPO (number = 49)**	***P *** **value**
Duration of ICU stay (days)	4.0 ± 5.1	3.5 ± 2.8	0.593
Duration of hospital stay (days)	14 (12 to 18)	15 (12 to 18)	0.695
Ventilatory care >48 hours	3 (6)	2 (4)	0.646
Postoperative inotrope use	17 (35)	13 (27)	0.381
Re-intubation	0 (0)	2 (4)	0.495
Re-operation	2 (4)	3 (6)	1.000
Postoperative atrial fibrillation	14 (29)	9 (18)	0.233
Stroke	1 (2)	2 (4)	1.000
Death	1 (2)	0 (0)	1.000

Values are mean ± SD or median (25th to 75th) or number of patients (%). EPO, erythropoietin.

None of the patients developed adverse complications related to EPO administration including thromboembolic events throughout the study period.

## Discussion

In this randomized controlled trial, we did not observe any beneficial influence of pre-emptive EPO administration on the incidence of AKI and the degree of renal injury following complex valvular heart surgery in patients at risk of AKI. In addition, EPO did not attenuate the postoperative increase in IL-6 and MPO levels.

AKI occurs in up to 50% of patients after cardiac operations depending on the chosen AKI definition or type of operation [1,17]. AKI is associated with increased resource utilization and poor prognosis [2]. Known predisposing risk factors for AKI include advanced age, congestive heart failure, anemia, diabetes mellitus and decreased basal renal function [1]. Yet, the predominant risk factor for AKI continues to be the use of CPB [5,6]. In the pathogenesis of postoperative AKI, renal ischemia-reperfusion injury and systemic inflammatory response likely play pivotal roles [1]. Owing to its unique circulatory characteristics, the renal medulla is especially vulnerable to ischemic insults, as it normally receives blood with low oxygen tension [18]. Thus, the inevitable alterations caused by CPB, such as hemodilution, renal vasoconstriction, embolism and systemic inflammatory response, may contribute to the development of AKI [6]. Indeed, patients undergoing valvular heart surgery requiring prolonged CPB were shown to be more vulnerable to AKI [19]. Despite continuing clinical efforts to diminish renal injury through abrogation of the pathways related to ischemia-reperfusion injury and inflammation, no intervention has conclusively proven efficacy.

EPO, an endogenous hormone mainly produced in interstitial fibroblasts of the renal cortex and outer medulla, is responsible for stimulating red blood cell development in response to hypoxia. In addition, EPO has pleiotropic organ protection properties and elicits cytoprotective responses in numerous tissues [9]. In recent years, mounting experimental evidence regarding the cardio-, renal- and neuroprotective effects of EPO against ischemia-reperfusion or inflammation-induced injury has been documented [20]. EPO may attenuate apoptosis by reducing oxidative stress and proinflammatory activation [8]. Despite its sound experimental background [8,9], only a few human studies have been performed regarding EPO’s renal protective effects and these studies have produced conflicting results [11-13,21].

In a pilot study performed in low-risk patients undergoing coronary artery bypass graft surgery (CABG), 300 IU/kg of EPO significantly decreased the incidence of AKI and the degree of sCr elevation [11]. The authors observed similar results in a previous study addressing the role of 500 IU/kg of preemptive EPO treatment as a blood conservation strategy in patients undergoing valvular heart surgery [13]. Since the patients who received EPO treatment received significantly less transfusion blood, the renal protective effect of EPO could not be clarified in that previous study. Clinical evidence regarding the role of EPO in the prevention of AKI in cardiac surgical patients who are at increased risk of developing postoperative AKI is lacking.

Contrary to our expectations, a pre-emptive single bolus of 300 IU/kg of EPO neither reduced the incidence of AKI nor attenuated the increase in biomarkers of renal injury in this study. Although sCr is widely used for assessing renal function, it has inherent limitations as a late indicator of renal dysfunction and an inaccurate detector of mild renal impairment [22]. Cystatin C is more precise in detecting early and mild renal impairment than sCr [23]. Likewise, NGAL discriminates patients with subclinical AKI accompanied by normal sCr [24] and is an effective predictor of AKI after CABG [25]. As we could not observe any differences in the levels of cystatin C or NGAL between the two groups in the current trial, EPO does not seem to provide any renal protection to the patient population in this study. Furthermore, the given dose of EPO did not exert any influence on the IL-6 and MPO levels in this study. IL-6 is a reliable marker of inflammatory cascade activation and is an indicator of poor outcomes [26], whereas MPO represents polymorphonuclear leukocyte accumulation [27]. Leukocyte infiltration is one of the key initiating processes of renal ischemia-reperfusion injury [28]. Both IL-6 and MPO were significantly attenuated with EPO in an experimental ischemic myocardial model [10,27] but not in this study.

There are several plausible explanations for the lack of benefit from EPO treatment. First, as our results indicate, the percentage of patients requiring renal replacement therapy was higher in the current trial compared with other studies [11,12]. Thus, the employment of a single preventive strategy to mitigate the development of AKI may not be enough to inhibit the numerous interconnections of signaling pathways that lead to renal injury. Multimodal strategies acting on various mechanisms may be required to mitigate the incidence of AKI in this high-risk group of patients. Second, the optimal dosage of EPO treatment to induce tissue protection has not been established clinically. In the present study, the dose of EPO (300 IU/kg) was decided based on a previous clinical study demonstrating the renal protective effects of EPO in low-risk patients undergoing CABG [11]. Although most of the patients in that study underwent off-pump CABG, CPB was reported to have no apparent influence on the pharmacokinetics of a single bolus of EPO [29]. The inconsistent results of the current study from that previous study might be associated with the different degree of the risk of the enrolled patients for postoperative AKI and the degree of inflammatory and oxidative stress between both studies.

Generally, higher doses of EPO are considered necessary for tissue protection than for erythropoiesis because of the lower affinity of the EPO heteroreceptor [9,30]. On the other hand, EPO’s organ protective effects at lower dosages (<500 IU/kg) have also been observed [31]. Similarly, some studies using higher doses of EPO have failed to show a beneficial effect [32]. Indeed, no difference in changes of urinary NGAL has been reported in a study that randomized post cardiac surgical patients up to 40,000 IU EPO, which is in accordance with the results of the current study [12]. Although used in a different clinical setting, doses up to 60,000 IU used in patients with myocardial infarction within four-hour reperfusion also could not provide any beneficial influence in terms of infarct size reduction [33]. Of note, the incidence of adverse cardiovascular events including death was significantly higher in patients who received EPO in that study, raising alarm against the use of higher doses of EPO.

Despite the organ protective effects observed in animal experiments, a recently increasing amount of negative evidence regarding the tissue protective effects of EPO has been documented in clinical trials. In terms of EPO’s anti-inflammatory effects, EPO did not suppress the systemic inflammatory response [12,26] and, instead, augmented the cytokine response to acute inflammation [34]. In accordance with those reports, no convincing trend in any of the serum levels of MPO and IL-6 was observed in the EPO group in the current trial. Of interest, one experiment in a porcine model suggested that EPO may protect against injuries related to direct ischemia but not injuries related to inflammation [32]. Considering that most experimental studies reporting the renal protective effects of EPO used a direct renal ischemia-reperfusion model, different mechanisms and degrees of renal injury in procedures using CPB without overt mechanical renal ischemia could also be responsible for the different results observed in clinical trials. As yet, the promising organ protective effects of EPO in experimental studies could not be consistently reproduced in clinical trials for numerous possible reasons that are beyond the scope of this study.

There are several limitations of this study. First, the sample size may not have been sufficient, as the actual incidence of AKI was lower than expected. Still, we did not observe any trend indicating a potential benefit of EPO, even with the sensitive biomarkers of renal injury known to detect subtle forms of renal dysfunction. Therefore, it seems reasonable to conclude that the given dose of EPO does not offer renal protection in the studied population. Second, more patients had DM in the EPO group, although these results were not statistically significant. DM was reported to mitigate the tissue protective effect of EPO, as well as various preconditioning strategies in animal studies [35-39]. There were no differences in mean blood glucose levels, renal biomarkers and the incidence of AKI between the groups even in patients without DM (data not shown). Moreover, DM as covariate in the linear mixed model for sCr to test whether it changes by the group and time interaction did not show any intergroup difference (*P* = 0.935); thus, the confounding effect of DM should be negligible. Third, the use of Voluven® might have confounded the results. Increasing evidence warns about the potentially harmful influence of hydroxyethyl starch solutions on AKI in critically ill patients [16,40]. However, the use of a limited amount of low molecular weight hydroxyethyl starch (less than 500 ml of Voluven® per day) does not seem to exert any influence on AKI as evidenced by the lack of association between the amount of Voluven® and changes in sCr in the current study (data not shown). Also, as both groups received a similar amount of Voluven® during the study period, its influence should be the same, if any should exist.

## Conclusions

Intravenous administration of a pre-emptive single bolus of 300 IU/kg of EPO did not decrease the incidence of AKI. It also failed to attenuate the increase in the serum levels of biomarkers of renal injury and Il-6 and MPO activation. A single preventive strategy, such as EPO, may not suffice in patients at increased risk of developing AKI after undergoing complex valvular heart surgery.

## Key massages

•Intravenous administration of a pre-emptive single bolus of 300 IU/kg of EPO did not decrease the incidence of acute kidney injury.

•It also failed to attenuate the increase in the serum levels of biomarkers of renal injury, such as sCr, cystatin C and NGAL.

•In terms of the anti-inflammatory effects of EPO, administration of a preemptive single bolus of 300 IU/kg of erythropoietin did not decrease IL-6 and MPO.

## Abbreviations

ACC: Aortic cross clamp; ACEi: Angiotensin-converting-enzyme inhibitors; AKI: Acute kidney injury; AKIN: Acute kidney injury network; ARB: Angiotensin II receptor blockers; ARF: Acute renal failure; AVR: Aortic valve replacement; BMI: Body mass index; CABG: Coronary artery bypass graft surgery; CCB: calcium channel blockers; CPB: Cardiopulmonary bypass; CrCl: Creatinine clearance; DM: Diabetes mellitus; eGFR: Estimated glomerular filtration rate; EPO: Erythropoietin; IL-6: Interleukin 6; MPO: Myeloperoxidase; MVR: Mitral valve replacement; NGAL: Neutrophil gelatinase-associated lipocalin; NYHA: New York heart association functional classification; POD: Postoperative day; pRBCs: Packed red blood cells; sCr: Serum creatinine.

## Competing interests

The authors declare that they have no competing interests.

## Authors’ contributions

JHK and YLK conceived the study design, collected, analyzed and interpreted the data, performed the statistical analysis and wrote the manuscript. JKS contributed substantially to interpreting the data and critically revised the manuscript for important intellectual content. JWS and YS contributed substantially to collecting and interpreting the data. HBK contributed substantially to collecting the data. All authors read and approved the final manuscript.
